# Validity and reliability of the Arabic version of the child perceptions questionnaire for 8–10-year-old children

**DOI:** 10.1007/s11136-020-02545-y

**Published:** 2020-06-10

**Authors:** Deem Al-Blaihed, Azza A. El-Housseiny, Nada J. Farsi, Najat M. Farsi

**Affiliations:** 1 Department of Pediatric Dentistry, Faculty of Dentistry, King Abdulaziz University, Jeddah, Saudi Arabia; 2 Department of Pediatric Dentistry, Faculty of Dentistry, Alexandria University, Alexandria, Egypt; 3 Department of Dental Public Health, Faculty of Dentistry, King Abdulaziz University, Jeddah, Saudi Arabia

**Keywords:** Children, Child perceptions questionnaire, Oral health-related quality of life, Reliability, Validity

## Abstract

**Purpose:**

To develop an Arabic version of the CPQ_8–10_ and test its validity and reliability for use among Arabic-speaking children.

**Methods:**

The 25-item professionally translated questionnaire included two global rating questions across four domains, which was assessed through a pilot study on 20 participants who were not included in the main study. Children (*n* = 175) aged 8–10 years were consecutively recruited: group I (*n* = 120) included pediatric dental patients, group II (*n* = 25) included children with orofacial clefts, and group III (*n* = 30) included orthodontic patients. Construct (convergent and discriminant) validity, internal consistency, and test–retest reliability were assessed using Spearman’s rank correlation coefficients, Cronbach’s alpha coefficient, and intraclass correlation coefficient, respectively. All children were clinically examined; 66 children completed the questionnaire a second time. A cross-sectional study design was employed.

**Results:**

CPQ_8–10_ scores and global ratings were positively correlated. CPQ_8–10_ scores were highest in group II, followed by groups I and III, respectively. CPQ_8–10_ scores were significantly higher in children affected with caries or malocclusion compared to unaffected children. Cronbach’s alpha was 0.95 and the intraclass correlation coefficient was 0.97.

**Conclusions:**

The Arabic CPQ_8–10_ was valid and reliable; therefore, it can be utilized with Arabic-speaking children in this age group.

## Introduction

Oral health is an imperative yet a frequently overlooked element that significantly alters the overall health and quality of life (QoL) [1]. Traditionally, oral health has been defined as the absence of disease [2]. However, this definition fails to consider the person’s values, understandings, and expectations [2]. Furthermore, existing definitions of oral health mostly lack to address all the domains and elements that are integral factors of oral health [2]. Therefore, the new definition acknowledges the versatile nature and attributes of oral health, which can be defined according to the World Dental Federation (FDI) as “Oral health is multifaceted, and includes the ability to speak, smile, smell, taste, touch, chew, swallow and convey a range of emotions through facial expressions with confidence and without pain, discomfort and disease of the craniofacial complex” [3]. Oral health-related quality of life (OHRQoL) reflects patients’ self-perception of their present oral health status in addition to its effect on their QoL [1]. There has been an increasing interest in evaluating the effect of oral conditions on individuals’ QoL, which resulted in the emergence of several evaluation instruments [4]. These instruments attempt to determine the extent that dental and oral disorders affect individuals’ daily lives [5].

The most frequently used measures among children are the Child Perceptions Questionnaire (CPQ) [6, 7], the Child Oral Impacts on Daily Performances [8], and the Child Oral Health Impact Profile [9]. These measures differ in dimensions, age of targeted children, and methods of reporting OHRQoL (either by the children themselves or by a representative). These questionnaires comprise a variety of oral conditions like dental caries, malocclusion, and craniofacial anomalies [10]. They were devised to measure the impact of oral health conditions on the daily lives of children and adolescents [10].

The CPQ offers a comprehensive assessment in understanding OHRQoL. It also offers an extensive perspective on oral diseases and disorders in children. Consequently, the CPQ has the capability to help determine necessary treatments and selected therapies, progress monitoring, and evaluate the outcomes of therapies for affected children in several contexts like research purposes, clinical practices, and formulation of new policies [7].

The CPQ for 8–10-year-old children (CPQ_8–10_) was developed and validated in Canada [7]. It showed good construct validity, excellent internal consistency, and acceptable test–retest reliability [7]. It is one of the most commonly used scales to detect OHRQoL [10]. It consists of 25 items distributed among 4 domains: oral symptoms, functional limitations, emotional well-being, and social well-being. It is self-reported by 8–10-year-old children using a 5-point Likert scale, and responses range from 0–4 for each item. Hence, total scores range from 0 to100, and higher scores indicate poorer OHRQoL [10].

The CPQ_8–10_ has been translated and validated in different languages such as Portuguese [4], Danish [1], Bosnian [11], Spanish [12], and Korean [13]; all of which revealed the scale to be valid and reliable for use among 8–10-year-old children. However, it has not been translated into Arabic; therefore, the aim of this study was to develop an Arabic version of the CPQ_8–10_ by translating the English version into Arabic and assessing its psychometric properties.

## Materials and methods

### Study design

A cross-sectional study design was employed following the Strengthening the Reporting of Observational Studies in Epidemiology (STROBE) recommendations in the conduct and dissemination of observational studies [14].

### Participants

A consecutive sample of 175 participants aged 8–10 years, who were seeking care at King Abdulaziz University Dental Hospital in Jeddah, Saudi Arabia, were recruited between December 2016 to January 2018. They were divided into three groups: group I consisted of 120 pediatric patients who were seeking dental care, group II included 25 pediatric patients with cleft lip and palate, and group III consisted of 30 orthodontic patients who were caries free and undergoing active orthodontic treatment.

Inclusion criteria included Arabic-speaking children, who had not received dental treatment in the past four weeks prior to study conduction, and with their central incisors and first permanent molars in occlusion to permit adequate assessment of the developing occlusion. Children with systemic and/or mental developmental disorders were excluded. Parents/guardians provided written consent prior to study commencement.

The sample size of group I was calculated using the Sauders and Huynh tables for estimation of sample size for reliability studies [15]. Given that the scale had 25 items, and it was assumed to be of moderate difficulty and variability—with a degree of precision of 0.05%—the minimum sample size required was 100; however, we increased that by 20% to compensate for any missing data. Group II included all patients with cleft lip and palate during the period of recruitment who attended the pediatric dental clinics. Lastly, group III included all orthodontic patients during the period of recruitment who attended the pediatric dental clinics.

## Ethical approval

This study was approved by the Research Ethics Committee of the Faculty of Dentistry, King Abdulaziz University, Jeddah, Saudi Arabia (no. 064-16).

### Measure

The original English CPQ_8–10_ contains two questions about children’s demographic data; i.e., sex and age [7]. It comprises 25 items distributed among 4 domains: oral symptoms (*n* = 5), functional limitations (*n* = 5), emotional well-being (*n* = 5), and social well-being (*n* = 10). The questions ask about the frequency of events in relation to children’s oral/orofacial condition arising in the previous four weeks. All 25 questions are measured using a 5-point Likert scale: “never = 0,” “once or twice = 1,” “sometimes = 2,” “often = 3,” and “Every day or almost every day = 4.” In addition, the scale also includes two global questions, which rate children’s oral health and the extent that their oral/orofacial condition impacts their overall well-being. Those responses were assessed with four-point Likert scales ranging from “very good = 0” to “poor = 3” and from “not at all = 0” to “a lot = 3,” respectively.

The English CPQ_8–10_ version was translated into formal Arabic using the well-recognized forward–backward technique, as recommended by Behling and Law [16]. Translation from English into Arabic was performed independently by two English speaking native Arabic speakers. Both translations were matched and discussed to develop the first version of the Arabic CPQ_8–10._ The Arabic translation was translated back into English independently by two other fluent bilingual investigators, who were blinded to the original English instrument. The backward translation was compared to the original English instrument to evaluate the conceptual and literal similarities.

#### Pretest technique

**Fig. 1:**
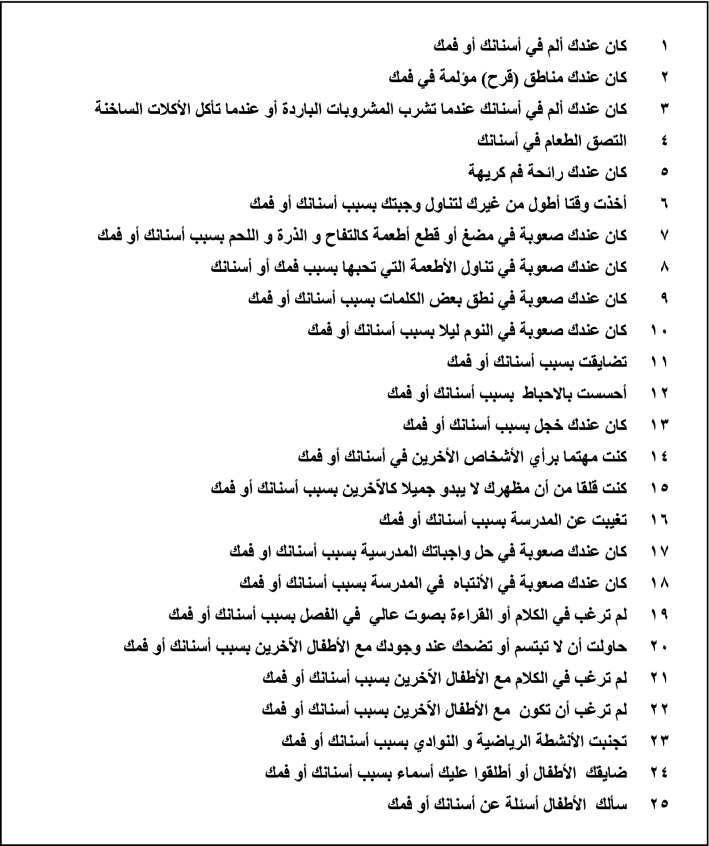
Arabic CPQ_8–10_-scale questions

Content validity is defined as “the degree to which the content of a health-related patient-reported outcomes (HR-PRO) instrument is an adequate reflection of the construct to be measured” [17]. After the first version of the Arabic CPQ_8–10_ was reviewed, it was evaluated for clarity and ease of administration through a pilot study with 20 native Arabic-speaking children (aged 8–10 years), who were not included in the main study, and who attended the pediatric dentistry clinic at King Abdulaziz University Dental Hospital, in order to assess the content validity. A one-to-one interview was conducted between the examiner and participant to assess each child’s understanding of the instructions, wording of the items and response options in order to evaluate the comprehensibility. Then, the children were asked to identify the misunderstood words. Following that, a discussion was organized between the researches in order to agree on the most easily understood words that maintained the same meaning. Consequently, minor wording revisions were made; for example, using “did you have” instead of “did you get”. Figure 1 illustrates the finalized Arabic version of the CPQ_8–10_.

### Procedure

Demographic data for the children were obtained from their parents in the dental clinic waiting area. Questionnaire administration was conducted via interviews with each participating child in the waiting room. Parents were instructed not to assist their children or interfere with the interview process. Then, clinical dental examinations were conducted in the dental clinics. Further, a subgroup of 66 children who had a second visit after 2 weeks completed the questionnaire again in order to assess the test retest reliability. To be eligible, they could not have received any dental treatment within those 2 weeks.

Prior to the conduction of the clinical examination, two pediatric dentistry residents completed calibration sessions using an agreed-upon rubric and were trained and calibrated at different times throughout the study—with 75–93% inter-rater reliability agreement for the “Decayed, Missing, Filled Teeth” (DMFT/dmft), and 90–100% inter-rater reliability agreement for the “Dental Aesthetic Index” (DAI).

Clinical data were collected using the World Health Organization’s criteria [18]. Clinical examinations were conducted on a dental chair using a mouth mirror and a blunt community periodontal index (CPI) probe (Screen probe Shepherd’s Hook, Nordent, USA). Caries experience was recorded using the DMFT/dmft index, which is the sum of decayed, missing, and filled teeth in permanent/primary dentition [18], using an examination chart. Malocclusion was classified according to the DAI [19], which is an international index that identifies occlusal traits and links clinical and aesthetic components mathematically to derive a single score that combines physical and aesthetic aspects of occlusion [20], according to the level of orthodontic treatment needs [21]. Orthodontic treatment needs were categorized into: “no or slight treatment needs” (DAI scores ≤ 25), “elective treatment needs” (DAI scores = 26–30), “highly desirable treatment needs” (DAI scores = 31–35), or “mandatory treatment needs” (DAI sores ≥ 36). After the examination, children were referred for treatment under the care of pediatric dental residents.

### Statistical analyses

The “COnsensus-based Standards for the selection of health Measurement INstruments” (COSMIN) approach was used for the definitions of the psychometric properties [17]. For each participant, the subscale domain scores and the total CPQ_8–10_ scores were calculated by adding the response scores. Lower scores indicated a better OHRQoL, while higher scores indicated a worse OHRQoL. All analyses were conducted using Stata 12.2 (StataCorp LP, College Station, Texas, USA). Significance was set at *P* < 0.05. The data were assessed for normality by visual inspection of a histogram and a quantile–quantile (Q–Q) plot, and by assessing results of Skewness and Kurtosis test for normality. The data were not normally distributed, so non-parametric tests were used.

### Validity

Construct validity was assessed, by hypothesis testing measurement property; which is defined as “The degree to which the scores of the HR-PRO instrument are consistent with hypothesis (for instance with regard to internal relationships, relationships to scores of other instruments, or differences between relevant groups) based on the assumption that the HR-PRO instrument validly measures the construct to be measured” [17]. Two types of hypothesis testing validity were assessed in this study, convergent validity and discriminant validity [17].

#### Convergent validity

Convergent validity refers to how close a scale score is related to other measures that should be theoretically related [22]. Convergent validity was determined by establishing the correlations between the scores for the total scale and each subscale (domain) with the overall well-being global ratings of oral health using Spearman’s rank correlation coefficients. Additionally, Wilcoxon rank sum tests were used to compare the means of the total scale and each subscale domain scores, with the dichotomized global ratings of oral health (good/very good and fair/poor) and overall well-being (not at all/a bit affected and sometimes/a lot affected), to test the hypothesis that individuals reporting poor oral health and negative well-being would have higher CPQ_8–10_ scores than would their counterparts, as we believe that participants who reported better global ratings of oral health scores would correspond with better CPQ_8–10_ scores. The global ratings were dichotomized, to allow sufficient number of participants in each category for statistical analyses.

#### Discriminant validity

According to Hubley, 2014, discriminant validity is “demonstrated by evidence that measures of constructs that theoretically should not be highly related to each other are, in fact, not found to be highly correlated to each other” [23]. We hypothesized that the level of distress of the three groups of children would be different, given the difference in the severity of the conditions. Therefore, children in group II would have the worst OHRQoL, followed by group I, then group III, in order to distinguish between groups of respondents that are not highly related to each other. So, discriminant validity was determined by comparing the scores in the three groups using Kruskal–Wallis tests, additionally, Wilcoxon rank sum test was used as a Post hoc test to compare each two groups.

We also examined the association between untreated dental caries and the total and subscale scores calculated, using Wilcoxon rank sum tests. The mean CPQ_8–10_ scores in children without dental caries (d/D = 0) were compared with the mean CPQ_8–10_ scores in children with dental caries in one or more teeth (d/D ≥ 1). Additionally, the association between the orthodontic treatment needs with the overall CPQ_8–10_ and subscale scores were calculated using Wilcoxon rank sum tests. The orthodontic treatment needs scores were dichotomized: no/slight/elective orthodontic treatment needs (≤ 30) and highly desirable/mandatory orthodontic treatment needs (> 30). It is hypothesized that children affected with dental caries would have worse OHRQoL than those without caries, and that children with more orthodontic treatment needs would have worse OHRQoL than those without or little orthodontic treatment needs.

### Reliability

Reliability is defined as the extent to which the scores of a scale remain unchanged under different circumstances, given that they were unchanged [17].

#### Internal consistency

Internal Consistency is the degree to which the different items in the instrument are related [17]. Internal Consistency was assessed using Cronbach’s alpha coefficient. The alpha value varies between 0 and 1. Cronbach’s alpha coefficient should be at least 0.7 to 0.8 in order to be considered satisfactory when comparing groups [24]. A low Cronbach’s alpha indicates a lack of correlation between the scale items, which means combining them to give an overall score is not meaningful. A high Cronbach’s alpha value indicates excellent correlation [10].

#### Test–retest reliability

Test–retest reliability was assessed on sixty-six children from group I, to ensure that the scores on repeated measurements over time do not change [17]. It was assessed using the intraclass correlation coefficient (ICC) in all three groups by determining the level of association between scores for the first and second rating of the total scale and subscale scores for the 66 participants who were interviewed twice.

## Results

### Pretesting results

The Arabic CPQ_8–10_ version was done by adjusting the translation of the following words: “ did you have “instead of “did you get” in questions 1, 2, 5, 7, 8, 9, 10, 13, 17 and 18 and by adjusting the words “felt” to replace “ did you have a feeling of …” in question 12. The most difficult concept that the children did not understand was the word “ulcer” Therefore, the phrase “sore spots” was added next to the word “ulcer” to clarify the meaning to the children.

Table 1 shows participants’ demographic characteristics. There were no missing data in the questionnaires. Table 2 presents the descriptive statistics of participants’ CPQ_8–10_ scores.

**Table 1:** Participants' demographic characteristics (*n* = 175)

	Total study *n* = 175 *n* (%)	Group I *n* = 120 *n* (%)	Group II *n* = 25 *n* (%)	Group III *n* = 30 *n* (%)
Sex				
Male	83 (47.4)	55 (45.8)	15 (60.0)	13 (43.3)
Female	92 (52.6)	65 (54.2)	10 (40.0)	17 (56.7)
Age				
8 years	64 (36.6)	42 (35.0)	12 (48.0)	10 (33.3)
9 years	55 (31.4)	37 (30.8)	7 (28.0)	11 (36.7)
10 years	56 (32.0)	41 (34.2)	6 (24.0)	9 (30.0)
Age, mean (SD)	9.0 (0.8)	9.0 (0.8)	8.8 (0.8)	9.0 (0.8)
Nationality				
Saudi	99 (56.6)	77 (64.2)	7 (28.0)	15 (50.0)
Yemini	49 (28.0)	26 (21.7)	10 (40.0)	13 (43.3)
Egyptian	11 (6.3)	5 (4.2)	4 (16.0)	2 (6.7)
Syrian	3 (1.7)	3 (2.5)	–	–
Afghani	1 (0.6)	1 (0.8)	–	–
Palestinian	12 (6.9)	8 (6.7)	4 (16.0)	–

Group I: children seeking dental care, group II: children with cleft lip and palate, group III: children receiving orthodontic care

*SD* standard deviation

**Table 2:** Descriptive statistics of the overall and subscale scores of the CPQ_8–10_ (*n* = 175)

	Number of items	Range of possible values	Range	Median	Mean (SD)	Floor effect (%)	Ceiling effect (%)
Overall scale	25	0–100	0–79	15	19.5 (18.4)	8.0	0
Scale domain							
Oral symptoms	5	0–20	0–18	5	5.8 (4.5)	12.0	0
Functional limitations	5	0–20	0–17	3	4.7 (4.8)	24.0	0
Emotional well-being	5	0–20	0–18	2	4.5 (4.8)	29.1	0
Social well-being	10	0–40	0–31	2	4.6 (6.8)	37.1	0

The participants of three groups were included in this analysis

*CPQ*_*8–10*_ Child Perceptions Questionnaire for 8–10-year-old children, *SD* standard deviation

### Convergent validity

Participants’ overall CPQ_8–10_ scores and the domain scores were positively correlated with self-reported assessments of the influence of oral conditions on everyday life; i.e., the global rating items. The Spearman correlation coefficients were all significant (*P* < 0.001). Positive, moderate, and statistically significant correlations between oral health global rating, overall well-being global rating, and the total scale were observed (*r* = 0.5 and 0.6, respectively). A similar direction was seen with all the domains; the correlation ranged from 0.4 to 0.5 for oral health rating, and from 0.5 to 0.6 for overall well-being rating (Data not shown).

Table 3 displays the descriptive statistics of participants’ CPQ_8–10_ scores with the dichotomized global ratings. The mean score for children reporting their oral health global rating as “fair/poor” was significantly higher than those reporting that their oral health rating was “very good/good.” Additionally, the mean score for children reporting that their overall well-being was affected “sometimes/a lot” by their oral or orofacial condition was significantly higher than those reporting that it was “not at all/a bit” affected. A similar direction was seen for the rest of the questionnaire domains.

**Table 3:** Descriptive statistics for CPQ_8–10_ scores by global ratings (construct validity-convergent validity) (*n* = 175)

	Oral health global rating					Overall well-being global rating				
	Good/very good *n* = 111		Fair/poor *n* = 64		*P*^†^	Not at all/a bit affected *n* = 100		Sometimes/a lot affected *n* = 75		*P*^†^
	Mean (SD)	Median	Mean (SD)	Median		Mean (SD)	Median	Mean (SD)	Median	
Overall scale	13.4 (12.3)	12	30.2 (22.2)	25	< 0.001	10.8 (10.4)	7	31.2 (20.3)	29	< 0.001
Scale domain										
Oral symptoms	4.3 (3.5)	4	8.3 (5.0)	8	< 0.001	4.0 (3.6)	3	8.1 (4.6)	8	< 0.001
Functional limitations	3.2 (3.8)	2	7.3 (5.3)	7	< 0.001	2.5 (3.2)	1	7.6 (5.0)	7	< 0.001
Emotional well-being	3.1 (3.9)	2	6.8 (5.2)	7.5	< 0.001	2.4 (3.4)	1	7.2 (4.9)	8	< 0.001
Social well-being	2.7 (3.8)	1	7.8 (9.2)	3.5	< 0.001	1.9 (2.8)	0	8.3 (8.6)	6	< 0.001

The participants of three groups were included in this analysis

*CPQ*_*8–10*_ Child Perceptions Questionnaire for 8–10-year-old children, *SD* standard deviation

^**†**^Wilcoxon rank sum test

### Discriminant validity

Table 4 presents the overall and subscale CPQ_8–10_ scores according to the three groups. Of the three groups, group II had the highest scores. The differences among the scores were statistically significant. All the subscales showed the same direction of the differences between the three groups of children, except for the comparison between groups I and II in the oral symptoms domain, and the comparison between groups I and III in the social well-being domain.

**Table 4:** Overall and subscale CPQ_8–10_ scores by clinical group (construct validity-discriminant validity) (*n* = 175)

	Group I		Group II		Group III		*P*^†^
	Median	Mean (SD)	Median	Mean (SD)	Median	Mean (SD)	
Overall scale	13	18.7 (17.1)^a^	32	34.2 (12.0)^b^	2	10.5 (21.3)^c^	< 0.001
Scale domain							
Oral symptoms	5	6.4 (4.5)^a^	7	6.8 (2.4)^a^	1	2.3 (4.4)^b^	< 0.001
Functional limitations	3	4.3 (4.1)^a^	9	9.8 (4.7)^b^	0	2.2 (4.7)^c^	< 0.001
Emotional well-being	2	4.0 (4.4)^a^	10	9.9 (3.0)^b^	0.5	1.8 (3.9)^c^	< 0.001
Social well-being	1	4.1 (6.5)^a^	7	7.7 (4.6)^b^	0	4.2 (8.7)^a^	< 0.001

The participants of three groups were included in this analysis

*CPQ*_*8–10*_Child Perceptions Questionnaire for 8–10 year old children, *Group I* children seeking dental care, *group II* children with cleft lip and palate, *group III* children receiving orthodontic care. CPQ_8–10_ score means sharing the same superscript alphabetical letter are not significantly different from each other (Wilcoxon rank sum test, P ≥ .05) and vice versa. SD = standard deviation

^†^Kruskal–Wallis test

Table 5 illustrates CPQ_8–10_ scores according to the dental caries status. There was a significant difference in the total and subscale scores of the Arabic CPQ_8–10_ between children without dental caries and those with dental caries in one or more teeth. Children with untreated decayed teeth had higher overall CPQ_8–10_ scores than did caries-free children (*P* < 0.001). The same direction of differences was observed in all the other subscale domains, with the mean scores being significantly higher in children with untreated decayed teeth compared to children without dental caries.

**Table 5:** Overall and subscale CPQ_8–10_ scores by dental caries (construct validity-discriminant validity) (*n* = 175)

	No caries *n* = 73		Caries *n* = 102		*P*^†^
	Median	Mean (SD)	Median	Mean (SD)	
Overall scale	4	11.6 (18.9)	19	23.3 (19.2)	< 0.001
Scale domain					
Oral symptoms	1	3.3 (4.4)	6	6.7 (4.2)	< 0.001
Functional limitations	0	2.4 (4.1)	5	6.0 (4.7)	< 0.001
Emotional well-being	0.5	2.4 (4.5)	4	5.2 (4.7)	< 0.001
Social well-being	0	3.6 (7.3)	2	5.8 (10.7)	0.004

The participants of three groups were included in this analysis

*CPQ*_*8–10*_ Child Perceptions Questionnaire for 8–10-year-old children, *SD* standard deviation

^†^Wilcoxon rank sum test

Regarding the CPQ_8–10_ scores according to the DAI, there was an association between the overall CPQ_8–10_ scores and severity of malocclusion. Children with highly desirable/mandatory orthodontic treatment needs had, on average, higher overall scores than did children with no/slight/elective orthodontic treatment needs (29.5 ± 15 vs. 18.1 ± 20.2, respectively). Additionally, the same direction of differences was observed in the subscale domains (*P* < 0.001). However, the oral symptoms subscale did not show significant differences between the children with highly desirable/mandatory orthodontic treatment needs and the children with no/slight/elective orthodontic treatment needs (6.3 vs. 5.7, respectively) (Data not shown).

### Reliability

The Arabic CPQ_8–10_, showed acceptable to excellent internal consistency. Cronbach’s alpha was 0.95 for the entire scale; and 0.78, 0.82, 0.86, and 0.92 for the oral symptoms, functional limitations, emotional well-being and social well-being domains, respectively. With regards to test–retest reliability, the ICC value was highest for the overall scale, 0.97 (95% CI 0.95–0.98). While for oral symptoms, it was 0.91 (95% CI 0.86–0.95), and 0.93 (95% CI 0.90–0.95) for the functional limitation. The ICC for emotional well-being and social well-being was 0.93 (95% CI 0.90–0.96), and 0.90 (95% CI 0.81–0.96), respectively (Data not shown).

## Discussion

This study examined the validity and reliability of the Arabic version of the CPQ_8–10_. The instrument had appropriate construct validity (convergent and discriminant), internal consistency, and test–retest reliability among Arabic-speaking children aged 8–10 years. Every time an instrument is used in a new context or with a different group of individuals, it is necessary to re-establish its psychometric properties [4]. This study showed that the psychometric properties of the Arabic version of the CPQ_8–10_ were suitable among this target group.

The study showed that overall CPQ_8–10_ scores were positively correlated with the global assessment of the influence of dental health on children’s everyday life. Furthermore, all the subscales showed the same direction of results in the three groups of children, confirming the relationships between the Arabic CPQ_8–10_ scores and global ratings. Specifically, the analysis confirmed that higher CPQ_8–10_ scores and subscale scores for each of the four domains were associated with poorer self-perceived oral and general health, which was similar to the findings associated with the original CPQ_8–10_ except for the correlation between the functional limitations and social well-being scores with the oral health rating in the original version [7]. The correlation rank coefficient is considered moderate to high according to a study [25]. Also, the findings of our study were higher than similar studies that were conducted [1, 4, 7].

In this study the three groups were analyzed together in order to assess the correlation of the CPQ_8–10_ scores with the ratings for oral health and overall well-being. However, we don’t believe that the results were affected because usually children with different oral conditions would be able to rate their oral health and the extent of which their oral/orofacial condition would impact their overall well-being in a similar manner regardless of the oral health condition. Analyzing different groups together was also observed in the original version [7].

However, our results contrasted those associated with the Danish version [1], which showed that the relationship between the global ratings of oral health and CPQ_8–10_ scores was low concerning oral symptoms. This can be possibly explained by 8–10-year-old children’s familiarity with oral symptoms like loose primary teeth, which might have less of an impact on their daily lives. In addition, our results were not in agreement with the Brazilian version of the CPQ_8–10_ [4], which revealed non-significant associations between global ratings of oral health and the social well-being and functional limitations subscales. This might be explained by the differences in children's understanding of oral health and well-being that may be affected by age-related experiences related to oral health in 8–10 year old children. As they reported many problems related to natural processes such as primary teeth exfoliation and spaces prior to permanent teeth eruption which might simultaneously affect their QoL.

This study also demonstrated the critical impact of child’s oral and orofacial condition on their functional, emotional, and social well-being, and that children can give psychometrically acceptable accounts of that impact; thus, it performs well as a valid measure. Similarly, the original English version [7], demonstrated a higher impact in children with orofacial conditions than among other children. However, the Danish version [1] showed that children with orofacial conditions reported CPQ_8–10_ scores similar to those reported by healthy children. This can be explained by the chronicity of the cleft lip and palate condition, which may have allowed time for the affected children to adapt to their situation. It would be hypothesized that the scores of the three groups would be different, as a result of the different clinical conditions and severity. Children with cleft lip and palate would have the worst OHRQoL as a result of the functional limitations and psychological implications associated with the condition, while it’s hypothesized that children with caries would have a better OHRQoL, as the degree of distress due to caries would be somewhat less than that of cleft lip and palate. Moreover, the group with the best OHRQoL would be hypothesized to be the orthodontic treatment group, as they usually have good oral health, no caries and their level of distress would be the least of the three groups.

Our study showed good discriminant validity. Participants’ overall scores were associated with untreated dental caries status in all subscales, especially in the oral symptoms, functional limitations, and emotional well-being subscales, which is similar to the findings from the Korean study [13]. The lesions in untreated dental caries could progress to become painful and distressing. Additionally, children’s experiences during the mixed-dentition period are related to physiological processes like dental eruption which can simultaneously affect their OHRQoL [4]. According to other studies, children with more severe caries would experience a greater impact on their OHRQoL [5, 26–28]. However, in the Brazilian study, only primary dentition showed a significant correlation with overall CPQ_8–10_ scores [4]. Moreover, the English CPQ_8–10_ did not demonstrate discriminant validity between the groups studied i.e., a dental caries group and a cleft lip and palate group [7]. The authors stated that this was likely because the children had previously received clinical and psychological treatment [7].

The present study also showed that the overall scores were positively associated with the malocclusion status in all the subscales except for the oral symptoms subscale. Oral symptoms are likely associated with pain due to the presence of untreated dental caries or mechanical and frictional trauma from orthodontic appliances. Another possible explanation is that malocclusion severity due to a cleft lip and palate is a congenital disorder, which allows the children time to adapt to their situation. Further, affected children likely complain from food impaction and halitosis more so than physical pain. It is noteworthy to mention that social functioning and experiences might be more likely to show variability over time than the physical and emotional effects of oral and orofacial conditions, especially for young children owing to their rapidly evolving dental and facial features. In agreement with most previous studies, the CPQ_8–10_ allowed us to discriminate between groups with known differences in dental health [1, 4, 13].

The internal consistency of a questionnaire shows whether all the items that make up the instrument are related to one another [17]. The Cronbach’s alpha coefficient should be at least 0.7 to be considered satisfactory when comparing groups [24]. For clinical applications, much higher values are required, with a minimum of 0.9 being desirable [24]. The overall Cronbach’s alpha in the present study was 0.95, indicating a very high overall internal consistency. However, the Cronbach’s alpha for the oral symptoms domain was only satisfactory. This can be explained by how oral symptoms such as “sore spots” may show variability over time owing to the healing of the offending lesions.

Our study’s internal consistency was higher in comparison to that of the original questionnaire (0.89) [7]. Moreover, the Cronbach’s alpha for the Arabic version of the CPQ_11-14_ was only satisfactory (0.81) [5], as was the Cronbach’s alpha for the Lebanese cross-cultural adaptation of the Arabic version of the CPQ_11-14_ (0.71) [29].

Concerning subscales, in comparison to other additional studies, the present study showed higher Cronbach’s alpha values (0.78–0.95) than did the Korean K-CPQ_8–10_ (0.57–0.85) [13], the Canadian version (0.63–0.89) [7], the Denmark version (0.57–0.82) [1], the Australian version (0.65–0.88) [30], and the Brazilian version (0.67–0.95) [4].

The second questionnaire was administered after two weeks to prevent memory recall and to minimize clinical changes [10]. The ICC is considered excellent when it is above 0.90, good when between 0.75 and 0.90, moderate when between 0.5 and 0.75, and poor when less than 0.5 [31]. Our results showed excellent stability of the questionnaire for both the total scale and the subscales (0.90–0.97), which can be explained by the brief time between both administrations. The original instrument [7] showed good reliability, except for the social well-being subscale, which showed variability in children’s social functioning and experiences over time.

In daily clinical situations, dental healthcare providers will most likely be concerned about oral symptoms when they assess patients’ oral health. As demonstrated in this study, subjective experiences should be given more weight and are as important as other clinical indicators when evaluating children’s OHRQoL. Our findings underline the value of considering broader aspects of children’s dental health rather than only the clinical indicators. Moreover, it is vital to gain insight into how oral conditions affect children’s daily functioning and future development.

This study had a few limitations. First, we used the DMFT guidelines to evaluate dental caries [18], which might create problems with underestimating dental caries that may be present proximally and could not be viewed without the aid of radiographs. Moreover, although the number of participants was small in two of the groups, we do not believe that it could have affected the results. Seeing that orofacial clefts are relatively rare, also not many children between the ages of 8–10 are treated orthodontically. Therefore, the participants recruited were the maximum number that could be collected during the timeframe. Further, the length of time the questionnaire took to complete was quite long for young children, even with the help of an interviewer; therefore, a short form of the Arabic CPQ_8–10_ may be useful among large populations.

The strengths of the study lie in that the psychometric properties were evaluated using the same method as the original CPQ_8–10_ study [7]. Moreover, our study included a larger sample size compared to the original (*n* = 175 vs. *n* = 68, respectively) to effectively evaluate measurement equivalence. In the present study, none of the children had received dental treatment, which is important in discriminating their QoL due to their oral condition rather than the dental treatment that the child was subjected to. Further, participants were consecutively recruited from clinical settings and they represented a wide range of Arabic nationalities. Moreover, the interview process for collecting the data prevented any external influence on children’s responses; thus, the study effectively reflected children’s own judgments and perceptions. Additionally, the data collected had no missing values.

In conclusion, the Arabic version of the CPQ_8–10_ was a valid and reliable instrument for measuring OHRQoL among 8–10-year-old Arabic-speaking children. The Arabic CPQ_8–10_ showed good convergent validity, discriminant validity, internal consistency, and test–retest reliability.

## Data Availability

Data cannot be publicly shared because of participants confidentiality, but it can be provided as needed.
